# Insight on Rosaceae Family with Genome Sequencing and Functional Genomics Perspective

**DOI:** 10.1155/2019/7519687

**Published:** 2019-02-19

**Authors:** Prabhakaran Soundararajan, So Youn Won, Jung Sun Kim

**Affiliations:** Department of Agricultural Biotechnology, National Institute of Agricultural Sciences, RDA, Jeonju 54874, Republic of Korea

## Abstract

Rosaceae is one of the important families possessing a variety of diversified plant species. It includes many economically valuable crops that provide nutritional and health benefits for the human. Whole genome sequences of valuable crop plants were released in recent years. Understanding of genomics helps to decipher the plant physiology and developmental process. With the information of cultivating species and its wild relative genomes, genome sequence-based molecular markers and mapping loci for economically important traits can be used to accelerate the genome assisted breeding. Identification and characterization of disease resistant capacities and abiotic stress tolerance related genes are feasible to study across species with genome information. Further breeding studies based on the identification of gene loci for aesthetic values, flowering molecular circuit controls, fruit firmness, nonacid fruits, etc. is required for producing new cultivars with valuable traits. This review discusses the whole genome sequencing reports of* Malus*,* Pyrus*,* Fragaria*,* Prunus*, and* Rosa* and status of functional genomics of representative traits in individual crops.

## 1. Introduction

Rosaceae consists of 100 genera and 3,000 species. It is one of the most economically important families which comprised the fruit, nut, ornamental, aroma, herb, and woody plants. Edible crops domesticated for human consumption in Rosaceae include apple, strawberry, pear, peach, plum, almond, raspberry, sour cherry, and sweet cherry. Though most of the choices are dietary based, some of the vital phytochemicals and antioxidants in fruits of Rosaceae have potential to inhibit cancer. For instance, ellagic acid abundant in strawberry, red raspberry, and arctic bramble was shown to prevent cell proliferation and induce apoptosis of cancer cells [1, 2].

Rosaceae consist of highly distinctive fruit types such as drupe, pome, drupelet, and achene. Conventionally, Rosaceae has been divided into four subfamilies based on the fruit types such as Rosoideae (several apocarpous pistils mature into achenes), Amygdaloideae/Prunoideae (single monocarpellate pistil mature into a drupe), Spiraeaoideae, (gynoecium consists of two or more apocarpous pistils mature into follicles), and Maloideae/Pomoideae (ovary is compound and inferior where floral receptacle is fleshy edible tissues) [3]. Recently, the phylogeny of Rosaceae has been divided into three basal groups based on nuclear and chloroplast loci, namely, Amygdaloideae, Rosoideae, and Dryadoideae [1]. Amygdaloideae has included the other subfamilies such as former Amygdaloideae (n=8) (plum, cherry, apricot peach, almond, etc.), Spiraeaoideae (n=9) (*Spiraea*,* Aruncus*,* Sorbaria*, etc.), and Maloideae (n=17) (apple, pear, cotoneaster, etc.). Rosoideae (n=7) includes* Fragaria*,* Potentilla*,* Rosa*, and* Rubus*. Dryadoideae (n=9) includes* Cercocarpus*,* Chamaebatia*,* Dryas*, and* Purshia*.

Exhaustive breeding on fruit trees offered different types of variety with variant alleles of genes controlling the key traits. To produce the sustainable cultivars we need to extend functional genomics studies in Rosaceae. As Rosaceae consists of highly distinctive types of fruits and diversified growth patterns, multiple genome models are required to improve the agronomic practices, produce high-yield and disease resistance varieties, overcome self-incompatibility, and reduce juvenile period, long-lasting postharvest self-life, tolerant to chilling (storage), firmness against transportation damage, and higher nutritional content and health benefit values. An emergence of next-generation sequencing (NGS) technologies revolutionize biological field with its feasibility to assemble and annotate any size and number of the genome(s) [4]. High-throughput genome sequencing offers the substantial advantages for the explicit understanding of genetics and genomics [5]. Recent breakthrough in the sequencing technologies and the availability of tools improve the accuracy of* de novo* genome sequencing. Unveiling the genome information gives us an invaluable insight into the epigenetic characteristics [6]. Genes responsible for traits of agronomic importance are rapidly identified and characterized with the forward and reverse genetics studies on many plants [4]. Genome-wide association studies (GWAS) characterize the functional role(s) of gene [5]. Genotyping-by-sequencing (GBS) and marker assisted selection (MAS) helps the precise breeding program [4]. Genomics provides huge amount of information in convenient manner for evolutional studies. Comparative analysis among diverse plant families helps to know about the evolutionary details of the gene(s)/plant(s) [7]. Candidate gene mapping in one species serves as a substrate for comparative analysis of other related species [5].

Therefore, this review will cover the progress of NGS of important commercial and model plants in Rosaceae such as apple, pear, strawberry, peach, sweet cherry, apricot, and rose. Brief information about the functional genomics studies conducted on critical key traits of the above-mentioned plants are also covered in this review.

## 2. Genome Assembly and Annotation

Genome-scale study gives rich candidate genetic resource to deciphering the functional and regulatory networks for growth and development. NGS is the perfect platform to know about the genomic information which has wide application in crop improvement and evolutionary studies. Genome sequencing details of apple, pear, strawberry, peach, and rose have been given in Table 1. Desirable key traits will be discussed in functional genomics section.

### 2.1. Apple

Apple fruit has higher nutritional values. For several centuries, humans consumed apple-based beverages such as ciders [8].* Malus *x* domestica* or* M. pumila* is the widely growing apple tree. Ancestor of domesticated* M. domestica* is* M. sieversii*. It is originated in Central Asia (Southern China). Wild* M. pumila* tree bearing smaller sized fruits is still covered 80% of Tian Shan Mountains. Microsatellite markers study showed that* M. domestica* is genetically similar to European crabapple* M. sylvestris* than to the Asian wild apple* M. sieversii* [9, 10].

So far three genomes have been released in apple. Firstly, Velasco et al. (2010) covered 81.3% (603.9 Mb) of* M* x* domestica* Borkh “Golden Delicious” genome. In that, 57,386 genes were identified. Almost 67.4% of* M *x* domestica* genome consists of repetitive sequences [11]. Secondly, Li et al. (2016) covered about 90% (632.4 Mb) of* M. *x* domestica* Borkh “Golden Delicious” genome. A total number of identified protein-coding and noncoding genes were 53,922 and 2,765, respectively [12]. Thirdly, Daccord et al. (2017) assembled genome of* M *x* domestica* Borkh “Golden Delicious doubled-haploid” line (GDDH13). Estimated genome size of GDDH13 is 651 Mb, from which 649.7 Mb (99.8%) was assembled. However, only 42,140 protein-coding genes and 1,965 nonprotein coding genes were identified in GDDH13 genome [6]. Major burst of transposable elements (TEs) happening around 21 MYA was correlated with the uplift of the Tian Shan mountains as well as the diversification of apple and pear [11]. Study on structural and functional evolution of genome cannot be completed without characterizing the TEs. Around 59.5% of the GDDH13 genome was covered with the TE elements. Most interestingly,* HODOR* (High-Copy Golden Delicious repeat), TE consensus sequences are present at about 22.3 Mb (3.6% of genome) [6].

### 2.2. Pear

Pear is one of the most important temperate fruit. It is originated in Western China. In spite of thousands of cultivars, based on the habituation,* Pyrus* species are divided into two major groups such as Occidental pears or European pears (*P. communis*) and Oriental pears or Asiatic pears (*P. bretschneideri*) [13]. Nevertheless commercially important cultivars were domesticated from the wide range of wild species; still pear cultivation faces challenges such as susceptibility to the pear scab, black spot disease, self-incompatibility, early ripening, short shelf life, firmness, sucrose content, grit/stone cells, color and odor of fruit, and inbreeding depression [14].

Recently 97.1% of* P. bretschneideri* Rehd. (Chinese pear) genome, i.e., 512.0 Mb (42,812 genes), has been annotated by Wu et al. [15]. Following it, 577.3 Mb of* P. communis* (European pear) was sequenced. It covered around 98.4% of genome containing 43,419 genes [16]. Pear is phylogenetically closer towards the apple [1]. Hence higher collinearity was existed between the chromosomes of pear and apple. Pear and apple divergence could have happened only 5.4-21.5 MYA [15]. Presence of repetitive sequence about 53.1% in* P. bretschneideri* [15] and 34.5% in* P. communis* [16] hampered the investigation of uncharacterized regions.

### 2.3. Strawberry

Strawberry comes under the category of soft fruit. It is widely attracted for its aroma, bright red color, texture, and taste. Preserved/processed strawberries are largely used for ice creams, milkshakes, chocolates, etc. It is considered to be difficult to propagate.

#### 2.3.1. *Fragaria vesca*

*Fragaria vesca* is a diploid species generally called woodland strawberry. It has unique characteristics such as day neutrality, nonrunning, and yellow colored fruits. It is self-compatible and has short generation time. It is indigenous to northern Eurasia and North America [17].

Small genome (240.0 Mb) of strawberry (*Fragaria vesca* “Hawaai4”) showed the absence of whole genome duplications. Though all members of rosids shared the ancient triplication, no evidence of whole genome duplication was found in* F. vesca*. About 99.8% (239.5 Mb) of genome was covered with identification of 33,264 genes [17]. Later, Darwish et al. done the reference based reannotation and assembly of woodland strawberry* F. vesca* “YW5AF7” genome [18]. Similar to the macrosyntenic relationships between pear and apple,* Fragaria *shared the synteny with* Prunus*. Lesser genome size of* F. vesca* could be mainly due to the lack of highly abundant LTR retrotransposons (< 2,100 copies). Based on the obtained genome sequences, 389 rosaceous conserved orthologous set (RosCOS) markers were developed in Rosaceae [19].

#### 2.3.2. *Fragaria x ananassa*

*F. *x* ananassa* is commonly cultivated species that play an important role in the strawberry production worldwide. Interestingly,* F. x ananassa* was reported as an accidental hybrid rose in France during mid-1700 between* F. chiloensis* (Chile) and* F. virginiana *(North American cultivar) [17].

Genome size of this octoploid species* F. *x* ananassa* was estimated between 708 Mb and 720 Mb.* F. *x* ananassa* shared the genome information with wild diploids such as* F. iinumae*,* F. nipponica*,* F. nubicola*, and* F. orientalis *and their genome size is 221 Mb, 208 Mb, 202 Mb, and 349.3 Mb, respectively. The octaploid genome,* F. *x* ananassa,* was assembled about 697.7 Mb, and its wild relatives are as follows:* F. iinumae *199.6 Mb (90.3%),* F. nipponica *206.4 Mb (99.2%),* F. nubicola *203.6 Mb, and* F. orientalis *214.2 Mb (61.3%) [20]. In total, the number of genes identified from* F. *x* ananassa* was 230,838. Protein-coding genes identified in wild relatives are 76,760 in* F. iinumae*, 87,803 in* F. nipponica*, 85,062 in* F. nubicola*, and 99674 in* F. orientalis*. About 47.1% (328.2 Mb) of* F. *x* ananassa* genome consists of repeats. In case of wild relatives, 31.7% (63.3 Mb) in* F. iinumae*, 25.5% (52.6 Mb) in* F. nipponica*, 24.5% (49.9 Mb) in* F. nubicola*, and 26.3% (56.2 Mb) in* F. orientalis *are repeat regions in genome [20].

### 2.4. *Prunus*

*Prunus* fruit has attractive bright shiny skin color, subtle flavor, and sweetness. It has long generation time and bigger plant size. It needs 3-5 years for flowering/fruit production from planting. Processed cherry product is sold worldwide.

#### 2.4.1. Chinese Plum and Japanese Apricot (*Prunus mume*)

*Prunus mume* is the first plant in Prunoideae subfamily to be sequenced. Domestication of* P. mume* could have started 3,000 years ago in China [21]. This woody perennial is considered as the first tree to be bloomed during the transition from winter to spring at lesser than 0°C [22].

Out of 280 Mb of the genome size, 237.0 Mb (84.6%) was sequenced. Totally 31,390 protein-coding genes were characterized in the* P. mume*. Genome of* P. mume* provides information about the 1,154 candidate genes involved in flower aroma, flowering time, and disease resistance. Assembled genome contains 106.8 Mb (45.0%) of repetitive sequences. Investigation of* P. mume* genome with the* Vitis vinifera*, paleohexaploid ancestor showed that 27,819 gene models aligned with its seven ancestral chromosomes. It is noteworthy that 2,772 orthologs' (78.1%) collinearity blocks were present in the* P. mume* genome (Table 1). Comparative analysis of* P. mume* chromosome with the Rosaceae ancestral chromosome showed that 4, 5, and 7 chromosomes of* P. mume* does not undergo any changes and they are direct Rosaceae ancient chromosomes such as III, VII, and VI, respectively [23].

#### 2.4.2. Peach (Prunus persica)

Peach is one of great fruit that provides vitamins, minerals, fiber, and antioxidant compounds. Peach fruit is also called nectarine due to smooth skin without fuzz or short hairs. Selection and domestication of peach could have started in Yangzi River valley, China, around 7,500 years ago [24].

Whole genome analysis of* P. persica* L. “Lovell” covered 224.6 Mb (84.7%) of genome (estimated total size 265 Mb) and represented 27,852 protein-coding genes. Repetitive sequences present in peach were estimated as 84.41 Mb (37.14%) which is lesser than the apple (42.4%) and grape (44.5%). 67.26 Mb (29.60%), 20.56 Mb (9.05%), and 17.14 Mb (7.54%) appeared as TEs, DNA transposons, and unclassified repeats, respectively [25]. Recently,* P. persica* “Lovell” double haploid genome version 2.0 was released with deep resequencing approach. Assembled genome of 227.4 Mb (85.8%) contains 26,873 genes [26].

#### 2.4.3. Sweet Cherry (*Prunus avium*)

*Prunus avium* generally called sweet cherry is an important drupe fruit in the Rosacea family. Sweet cherry is used for human consumption and wild cherry trees for wood which is also called mazzards. Sweet cherry and sour cherry are the most commercial and edible crops in* Prunus* genus [27].

Genome size of* P. avium* is about approximately 350 Mb. Shirasawa et al. (2017) assembled about 77.8% (272.4 Mb) of the* P. avium* “Satonishiki”. About 43.8% (119.4 Mb) of the* P. avium* genome were covered with the repetitive sequences. Among the 119.4 Mb of repeats, 85.1 Mb of repeats are unique to* P. avium* “Satonishiki”. Identified genes clustered with the* P. persica*,* P. mume*,* M. domestica*, and* F. vesca*. 75,627 genes clusters are formed. 3,459 clusters (4,535 genes) from* P. avium* are present in all the investigated species and 16,151 clusters (21,642 genes) were found only in the* P. avium* with the absence of 869 clusters [28].

### 2.5. Rose

Roses are one of the most essential ornamental plants worldwide. Ornamental value of rose enjoyed since the dawn of civilization. Cultivation of roses traced back to 3000 years ago. It consists of 200 species and most of them are polyploid. It has also been cultivated for its cosmetic values such as perfumes and antiques and also some of the phytochemicals of roses have high therapeutic values. Rose hips can be used to cure osteoarthritis [29].

#### 2.5.1. *Rosa chinensis*

*Rosa chinensis* is one of the important pot-type rose cultivars. Recently, Raymond et al. (2018) sequenced the whole genome of* R. chinensis* “Old Blush” and resequenced the major genotypes contributed for rose domestication. Totally, 503 Mb (97.7%) of the genome was assembled. Genome results comprised 36,377 protein-coding genes, 3,971 long noncoding RNAs, and 207 miRNAs. In the genome TEs were present about 67.9%. From that, 50.6% were identified as long-terminal-repeat retrotransposons [30]. From the doubled-haploid rose line of* R. chinensis* “Old Blush” (“HapOB”) about 90.1 to 96.1% (512 Mb) of genome was assembled. About 466 Mb was anchored to seven pseudo-chromosomes and the remaining were assigned to the chromosome 0 (Chr0). Totally 44,481 genes were identified including 39,669 protein-coding and 4,812 noncoding genes. Repeats covered about 279.6 Mb of genome [31].* Rosa *and* Fragaria* genomes shared the eight chromosomes of ancestral Rosaceae with one chromosome fission and two fusions. Divergence of* Rosa*,* Fragaria*, and* Rubus* could have occurred within a short period [30]. Synteny analysis showed that chromosomes 1, 4, 5, 6, and 7 of* R. chinensis* have higher collinearity with chromosomes 7, 4, 3, 2, and 5 of* F. vesca*. Interestingly, chromosomes 2 and 3 of* R. chinensis* were detected as reciprocal translocation with chromosomes 6 and 1 of* F. vesca* [31].

#### 2.5.2. Rosa multiflora

*Rosa multiflora* is a five-petal plant belongs to the section* Synstylae*. It is native to the eastern Asian regions [32].* R. multiflora* was used for breeding purpose to the modern roses. Especially, its resistance locus (*Rdr1*), tolerance against powdery mildew was introgressed with the* R. hybrida* [33].

Genome size of* R. multiflora* was estimated as 750 Mb and about 711 Mb was sequenced. Assembled genome was characterized with 67,380 genes (54,893 complete genes and 12,487 partial genes). Repeat regions covered 56.4% (417.2 Mb) of assembled genome. Out of 18,956 gene clusters in* R. multiflora* 1,287, 904, and 241 clusters were shared with the* F. vesca*,* P. persica*, and* M* x* domestica*, respectively.* R. multiflora* shared more number of gene clusters with the* F. vesca* than the other two plants of Rosaceae. However, unique gene clusters and genes of* R. multiflora* are 2.5 (3,482 of* R. multiflora* and 1,397 of* F. vesca*) and 3.3 (14,663 of* R. multiflora* and 4,482 of* F. vesca*) times higher than the* F. vesca*, respectively [34].

## 3. Functional Genomics

### 3.1. Fruit Development and Sucrose Metabolism in Apple

Pome is a unique nature of false fruit formation from the basal part of sepals and receptacles. Velasco et al. (2010) suggest that pome could have evolved recently from Maleae specific WGD which could be a major factor contributing to apple development and its specificity [11]. Genes encoding for like-hetero chromatin protein 1 (LHP1) such as* MdLHP1a* and* MdLHP1b* regulate the flowering time of apple [35].* Flowering locus T1* (*MdFT1*) can promote flowering whereas* terminal flower* (*MdTFL1* and* MdTFL2*) expressed in the vegetative part could repress flowering and maintains the vegetative meristem identity [36]. Soon after fertilization, higher expression of two* cyclin-dependent kinase* (*CDK*)*b *genes and one* cyclin-dependent kinase regulatory subunit *(*CKS*)* 1* indicates the active cell division of fruits [37]. Transcription factors such as Agamous (AG), Fruitfull (FUL), AG-like (AGL)1/AGL5, Spatula (SPT), Crabs Claw (CRC), and Ettin (ETT) regulate the carpel identity and differentiation [38]. Microarray data on apple reported that SPT, ETT/Auxin Response Factor (ARF) 3, FUL/AGL8, and CRC transcripts were abundant during the fruit enlargement stage. However, most of their expressions are downregulated in cell division stage [39]. In apple, fruit development-related gene families such as* MADS-box* genes, carbohydrate metabolism, sorbitol assimilation, and transportation were expanded more than the cucumber, soybean, poplar,* A. thaliana*, grape, rice,* Brachypodium*, sorghum, and maize [11]. Expression of* α-expansin* (*α*-EXP) was detected only during the cell expansion phase of apple [39]. MdMADS2.1 and MdMADS2.2, orthologous to FUL-like genes in* A. thaliana,* were progressively involved in the fruit developmental process. Among two candidate genes, MdMADS2.1 was closely associated with fruit flesh firmness [40]. ARF106 gene expressed during cell division and cell expansion stages is consistent with a potential role in the control of fruit size [41]. Methylation of DNA plays an essential role in the fruit size [12]. Comparative study between the bigger size apple (Golden Delicious) and smaller size apple (GDDH18) showed that twenty-two genes found as responsible for small size have lesser methylation in the promoter region [6].

After pollination, the small amount of starch present in the floral buds starts to metabolize. Conversion of carbon to sucrose was mediated by the tonoplast monosaccharide transporters (TMTs),* MdTMT1* and* MdTMT2.* Expansion of fruit cells is associated with the starch accumulation. Higher expression of* sorbitol dehydrogenase *(*SHD*),* cell wall invertase *(*CIN*),* neutral invertase *(*NIN*),* sucrose synthase *(*SS*),* fructokinase *(*FRK*), and* hexokinase *(*HK*) indicates the metabolization of sorbitol and sucrose [42]. In the early period of cell expansion, starch accumulation was higher, and it starts to decline in the later phase [11]. Transcript of SS genes in apple is correlated with the starch accumulation [39].* Sorbitol dehydrogenase* (*SDH*) converts carbohydrate into fructose. Nine* SDH* genes were identified in apple fruit [43]. In young fruit,* MdSDH1* expression was higher than in mature fruit [42]. Other genes significantly upregulated during ripening stage are* isopentenyl pyrophosphate* (IPP)* isomerase*,* catalase *(CAT)*, histone 2B* (*H2B*), and the* ripening-inhibitor* (RIN)* MADS-box gene* [39]. During the ripening process, a decrease of starch synthesis is vice versa with the sugar level [44]. Expression profiles of* sucrose-phosphatase phosphatase *(*SPP*) and* sucrose-phosphate synthase* (*SPS*) were active in the ripening stage [42], suggesting that these enzymes may be involved in starch degradation pathway.* Polygalacturonase 1* (*MdPG1*) and aminocyclopropane-1-carboxylate oxidase (*MdACO1*) were involved in the fruit softening and ethylene biosynthesis in apple, respectively [45]. Decrease in the expression of* PG1 *alters the firmness, tensile strength, and water loss in apple M x domestica fruit [46]. Meanwhile,* MdFT1*,* MdACS1 *(*1-aminocyclopropane-1-carboxylic acid synthase*),* MdACO1*, and* MdExp7* are regulating the fruit softening. Among them,* MdExp7* and* MdACO1* control firmness in apple [45]. Gene coding for MYB TF in apple,* MdMyb1,* increases the anthocyanin content and is responsible for the red skin color [47]. Acidity in apple is due to the malic acid, and* mama* recessive gene is responsible for low acidity [48].

In apple, fruit size, sugar content, and palatability are essential qualities determining its marketability. Knowledge of genes governing the fruit quality could be essential for screening better lines/genotypes for breeding.

### 3.2. Lignin Metabolism and Stone Cell Formation in Pear

Stone cell content is the main quality determinant of pear fruit. Deposition of lignin on the primary cell wall of parenchyma cell followed by the secondary sedimentation on a sclerenchyma cell forms the stone cells. Majority of stone cells present in pear is branchy sclereids comprised lignin and cellulose. Lignins are synthesized by two ways, one starts with p-coumaric acid and second with phenylalanine precursor to cinnamic acid and then p-coumaric acid. Other forms of lignin monomers are caffeic acid, ferulic acid, 5-hydroxy-ferulic acid, and sinapinic acid [49–51]. Finally, monomers are polymerized to form lignin products. Monomers of lignin are categorized into three types, syringly lignin (S-lignin), guaiacyl lignin (G-lignin), and hydroxyphenyl lignin (H-lignin). From the gnome analysis, a total of 66 lignin synthesis-related gene families were characterized in* P. bretschneideri*. It signifies the high demand for lignin synthesis in pear [15]. In “Dangshan Su” pulp, milled wood lignin was identified as guaiacyl-syringyl-lignin. It was observed that “Dangshan Su” lignin possesses more guaiacyl units than the syringyl units [49].* Hydroxycinnamoyl transferases* (*HCT*) play a significant role in the lignin synthesis [52]. Accumulation of G-lignin and S-lignin is interrelated with the* HCT* expression especially at early fruit developmental stage [15].

**Figure 1:**
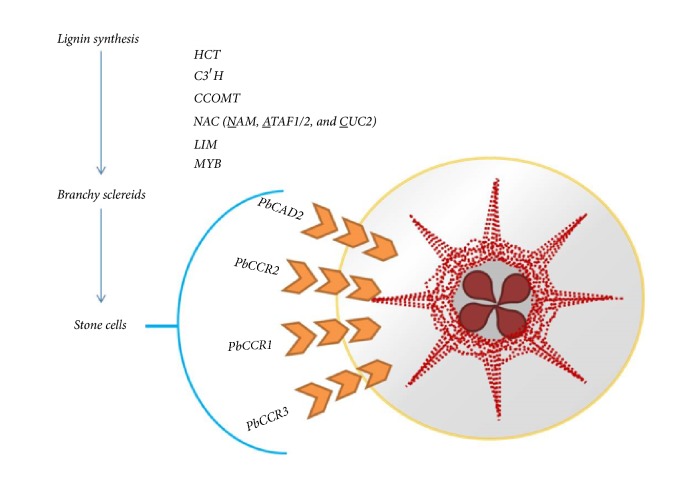
Simple heuristic representation of genes/transcription factors involved in lignin synthesis and stone cell formation in pear fruit.* Pb*,* Pyrus bretschneideri*;* hydroxycinnamoyl transferase*,* HCT*;* p*-coumaroyl-shikimate/quinate 3′-hydroxylases,* C3*′*H*;* caffeoyl-CoA O-methyltransferase*,* CCOMT*; NAM, ATAF1/2, and CUC2, NAC; Lin11/Isl1/Mec3, LIM; myeloblastosis, MYB;* cinnamyl alcohol dehydrogenase*,* CAD; *and* cinnamoyl-CoA reductase*,* CCR*. Red colored dots represent the stone cells.

Cinnamoyl-CoA reductase (CCR) and cinnamyl alcohol dehydrogenase (CAD), belonging to medium-or-short-chain dehydrogenase/reductase, are key enzymes for lignin monomer synthesis [53]. Totally 31* CCR*s and 26* CAD*s genes were identified in* P. bretschneideri* “Dangshan Su”. All members of* CCR* and* CAD* identified in* P. bretschneideri* are not involved in the lignin biosynthesis [54]. Among them,* PbCAD2*,* PbCCR1*,* PbCCR2*, and* PbCCR3* were identified to participate in the lignin synthesis of stone cells [15]. NAC (NAM, ATAF1/2, and CUC2) and LIM (Lin11/Isl1/Mec3) are an important TF influencing the lignin pathway [55, 56]. Most of the* CCR* and* CAD* members present in the pear possess SPL (squamosal promoter binding-like) light-responsive element on their upstream. Functions of* PbCCR* and* PbCAD* are related to the light signaling. Presence of MYB-binding AC* cis* elements in some promoter of the* PbCCR*s suggested that phenylpropanoid metabolism of lignin synthesis was regulated by MYB transcription factors. Similarly, TGACG-motif on some PbCCRs and all PbCAD's promoter regions revealed their involvement in the abscisic acid, jasmonic acid, and methyl jasmonic acid metabolism [15]. The pictorial illustration of genes/TFs required for the lignin synthesis as well as stone cell formation is mentioned in Figure 1.

There are many internal and external factors involved in the stone cell formation of pear. Identification of candidate genes of lignin biosynthesis and stone cell formation will be very much useful to improve the cultural practices for producing pear fruits with different palatable level of stone cells.

### 3.3. Fruit Aroma and Softness in Strawberry

Strawberry is widely appreciated for its delicate flavor, aroma, and nutritional value. Aroma of strawberry is due to esters, alcohols, aldehydes, and sulfur compounds. Hundreds of volatile esters have been correlated with strawberry ripening and aroma [57]. Volatile esters are the major constituents of floral scent. Wild species such as* F. vesca* and* F. virginiana* have much stronger aroma than the cultivated types. Compared to the regular octoploid strawberry, unique phenolic compounds were found in* F. vesca* fruits, such as taxifolin 3-*O*-arabinoside and peonidin 3-*O*-malonylglucoside [58].* Pinene synthase* (*PINS*) is primarily expressed in wild strawberry while insertional mutation reduced its expression in cultivated species.* F. vesca *contains high amounts of ethyl-acetate and lower methyl-butyrate, ethyl-butyrate, and furanone levels.* F. nilgerrensis* possesses higher ethyl-acetate and furanone but lower methyl-butyrate and ethyl-butyrate. Hybrids between* F. vesca* and* F. ananassa* have intermediate contents of fragrance and aroma related compounds while crosses between* F. nilgerrensis* and* F. ananassa* more closely resemble* F. nilgerrensis *[17]. Volatile compounds found to be responsible for general strawberry smell are 2, 5-dimethyl-4-hydroxy-3(2H)-furanone, linalool, and ethyl hexanoate. Nevertheless, ethyl butanoate, methyl butanoate, *γ*-decalactone, and 2-heptanone are represented as cultivar specific aroma compounds [59]. O-methyltransferase of strawberry (*FaOMT*) is vital for the biosynthesis of vanillin and furaneol [60].* Alcohol acyltransferase *(*AATs*) in strawberry (*SAAT*) is involved in the last step of volatile esters synthesis and vital for flavor biogenesis in ripening fruit.* SAAT* catalyzes esterification of an acyl moiety from actyl-CoA to alcohol [61]. Strawberry* quinone oxidoreductase* (*FaQR*) is required for the biosynthesis of furaneol. Furaneol and its methoxy derivative (methoxyfuraneol and mesifuran) are catalyzed by OMT. All three furaneol compounds are highly accumulated during fruit ripening stage [62]. Though two types of* pyruvate decarboxylase* (*PDC*) were identified in strawberry, only* FaPDC1* was induced during fruit ripening [63].

Strawberry is highly perishable even with controlled atmospheric storage. Higher proportion of fruit last occurred due to its softness and sensitivity to fungal disease. Red colored strawberry showed the higher level of anthocyanin-related transcripts [64].* FcMYB1* could regulate branching-point of the anthocyanin/proanthocyanidin biosynthesis.* FaWRKY1* mediate defense response and* FaPE1*, encoded for pectin methyl esterase, are conferred at least with a partial resistance of ripened fruit against Botrytis cinerea [65, 66].* Polygalacturonase 1* of* F *x* ananassa* (*FaPG1*) is critical for fruit softening [67]. In strawberry fruits* beta-D-glucosyltransferase *(*FaGT*) correlated with the relevant phenylpropanoid glucosides [68].* D-xylose reductase (FaXyl1)* and* beta-xylosidase* activity were higher in “Toyonaka” (soft) than in the “Camarosa” (firm) showing the correlation between* FaXyl1* expression and fruit softening [69]. Fruit-specific* rhamnogalacturonate lyase 1* (*FaRGLyase*) is involved in the firmness and postharvest life [70]. A lesser activity of beta-galactosidase (*βGal*) and* βXyl* activity were correlated with decreased fruit firmness in* F. chiloensis* and* F. × ananassa*, respectively [71]. Expression of* FaCCR* is higher in soft fruit cultivar (Gorella) whereas* FaCAD* is higher in firm fruit cultivar (Holiday) [72]. Expression of five* expansin* genes (*FaEXP1*,* FaEXP2*,* FaEXP4*,* FaEXP5*, and* FaEXP6*) was studied in cultivars with different firmness “Selva” (hard), “Camarosa” (medium), and “Toyonaka” (soft). Higher level of* FaEXP1*,* FaEXP2*, and* FaEXP5* expression was found in fruit with less firmness (“Toyonaka”) than the other two cultivars (“Selva” and “Camarosa”). Fruit firmness is identified to be associated with pectate lyase (*FaPel1*) identified. Expansin activity was characterized by cell wall modification [73]. Polysaccharides were modified by five different genes such as* FasPG*,* FaPG*-like,* FaPel1*,* FaPel2*, and* FaEXP2 *[74].* Sorbitol dehydrogenase* (*FaSDH*) and* sorbitol-6-phosphate dehydrogenase* (*FaS6PDH*) genes are involved with the sorbitol synthesis in leaves, fruits, and shoot tips [75].* SEPALLATA *(*SEP*)*4-like* gene* FaMADS9* is responsible for the fruit ripening [76].

Apart from the aesthetic and taste, mechanisms of flowering and its response to the light signaling in strawberry need to be studied in detail. Cultivars with continuous flowering and growing under minimal light energy are beneficial for the strawberry growers as most of the commercial cultivation is carried out in the controlled greenhouse.

### 3.4. Early Blooming and Fruit Ripening in* Prunus*

**Figure 2:**
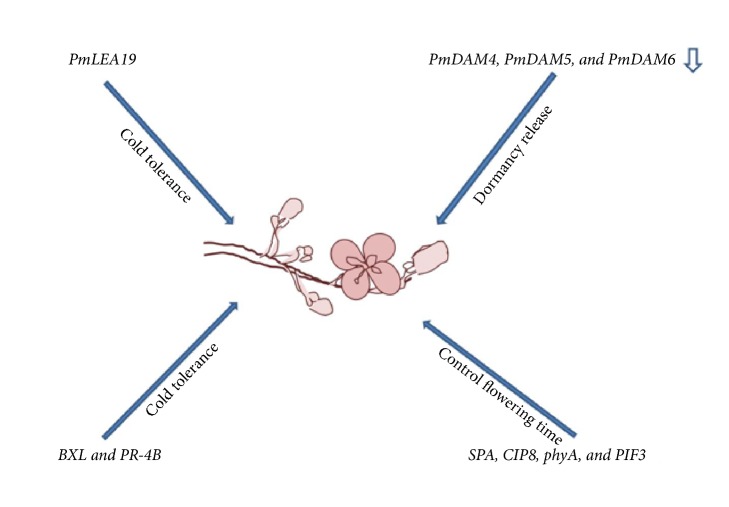
Factors involved in the early blooming of Chinese plum/Japanese apricot.* Prunus meme, Pm*;* late-embryogenesis-abundant*,* LEA*;* dormancy associated MADS*,* DAM*;* β-D-xylosidase, BXL; pathogen-related protein 4b, PR-4B*;* suppressor of phyA*,* SPA; COP1 interacting protein8*,* CIP8; phytochrome A, phyA; and phytochrome interacting factor3*,* PIF3*. The lower arrow represents downregulation.

*Prunus* is the first plant to bloom in later winter/early spring. So, it is the best model plant to study early flowering as well as chilling tolerance. Dehydrins are known as 2 or D-11 family late-embryogenesis-abundant (LEA) proteins. They play a vital role in plant growth and cold tolerance [77]. In* P. mume*, 30 LEA genes were characterized and classified into eight groups LEA1, LEA2, LEA3, LEA4, LEA5, PvLEA18, dehydrin, and seed maturation protein. Out of 30 identified genes, 22 were expressed in flowers, and 19 were induced by* abscisic acid* (ABA) treatments [78]. Molecular cloning of* PmLEA8*,* PmLEA10*,* PmLEA19*,* PmLEA20*, and* PmLEA29* showed that, except* PmLEA8*, all other genes enhanced the freezing-tolerance. Interestingly, among all cold-resistant* LEA* gene members studied, only* PmLEA19* were upregulated four times when the branches of* P. mume* were exposed to 4°C [79]. Downregulation in* P. mume dormancy associated MADS *(*PmDAM*)* 4*,* PmDAM5*, and* PmDAM6* expression releases the endodormancy [80]. Among the six* DAM* genes, except* PmDAM3*, all other genes are responsive to the photoperiod and seasonal (cold) responses [81]. In* P. persica* from six identified* DAM* genes,* PpDAM5* and* PpDAM6* were characterized to be involved in the lateral bud dormancy breakage [82].* DAM5* and* DAM6* were identified as homologous to* Short Vegetative Phase* (SVP)/*AGL 24* in* A. thaliana*. Both* SVP* and* AGL-24* are required for floral meristem identity [83]. AGL24 is well known for promoting early flowering and floral transition in plants [58]. Transcriptome analysis between cold sensitive (“Morettini”) and cold tolerant (“Royal Glory”) cultivars in* P. persica* showed that* β-D-xylosidase (BXL)* and pathogen-related protein 4b* (PR-4B)* were significantly expressed only in resistant variety [84]. Other candidate genes identified as required to control flowering time are* suppressor of phyA (SPA)*,* COP1 interacting protein8 (CIP8)*,* phytochrome A (phyA)*, and* phytochrome interacting factor 3 (PIF3)* [85].  Figure 2 demonstrates the important key genes involved in cold tolerance, early blooming, and flowering time control of* P. mume*. Higher number of aesthetic properties related genes such as* benzyl alcohol acetyltransferase* (*BEAT*) (34) are identified in* P. mume*. Only, 16 in* Malus* x* domestica*, 14 in* F. vesca*, 4 in* Vitis vinifera*, 17 in* P. trichocarpa*, and 3 in* A. thaliana *of* BEAT* genes were identified. Therefore, in* P. mume BEAT* genes are considered as key factor to determine its exclusive floral fragrance [23].

In Prunus,* UDP-glucose-flavonoid-3-O-glucosyltransferase (UFGT)* expression was higher during the initial period and it is reduced on the developmental process. During the ripening process,* MYB10, MYB123*, and basic-helix-loop-helix* (bHLH3)* were upregulated whereas* MYB16* and* MYB111* were downregulated. Higher anthocyanin and Proanthocyanidin levels were correlated with the* MYB10* and* MYBPA1*, respectively. Stimulation of TFs is responsive for the development and external stimuli [86]. Gene encoding* ethylene-responsive transcription factor* (*ERF*)*4* is necessary for the fruit maturity [87]. Though 74* EFR* genes were predicted in the peach genome, only one copy of* ERF4* has existed. Therefore,* ERF4 *is vital to control the fruit maturity and ripening in peach [85]. An initial stage of fruit has higher aldehyde and alcohol production whereas later stages have lesser content which is correlated with the ester production. Abundance of alcohol dehydrogenase (*ADH*) and* lipoxygenase *(*LOX*) gene is constant in the fruit development stages. Expression of* AAT* was sharply increased in the later stage of harvest [88]. Rapid softening of fruits was related to the ethylene production in* P. persica*. It is correlated with expression of* Pp*ACS1 (*1-aminocyclopropane-1-carboxylic acid synthase*) [89]. Ripened sweet cherry* P. avium* has unique fragrance. In the sweet cherry (“Hongdeng”, “Hongyan”, and “Rainier”) 97 volatile compounds were identified. Alcohols and terpenes were the predominant components of bound volatiles. Benzyl alcohol, geraniol, and 2-phenylethanol are the major bound volatile constituents. Free volatile compounds majorly present in sweet cherry are hexanal, 2-hexenal, 2-hexen-1-ol, benzyl alcohol, and benzaldehyde. Free volatiles are responsible for floral aroma and bound volatiles involved in fruit freshness. Depending on the level of free and bound volatiles, aroma and glycosidically bound compounds aroma and fruit firmness were varied between the cultivars of sweet cherry [90].

In* Prunus*, apricot, peach, sweet cherry, and sour cherry are widely used for human consumption. Comparative genomics study between the species offer the candidate gene to produce hybrids with more preferable qualities.

### 3.5. Blooming and Scent Pathways in Rose

Continuous flowering (CF)/recurrent blooming (RB) genotypes flowers in all favorable seasons, whereas once‐flowering (OF) genotypes only flowers in spring. Recurrent blooming is an important trait required by breeders.* R* x* hybrida* “La France” was the first hybrid combined with the growth vigor of European species and recurrent blooming of Chinese species. It has the complex genetic pool combination of three ancestral genotypes such as Cinnamomeae, Synstylae, and Chinenses. Insertion of TE in the* TFL1* encodes gene* Ksncopia* (*KSN*) was found as responsible for the recurrent blooming of “La France” [30]. Previously, Horibe et al. (2013) reported that* KSN* gene regulates the CF behavior of* R. rugosa *[91]. Wang et al. (2012) studied recurrent flowering character and the expression patterns of TFL1 homologs in* R. multiflora, R. rugosa, R. chinensis*, and other species/cultivars. Among the three orthologs, RTFL1c was highly expressed at all four flowering stages in* R. multiflora* and* R. rugosa* (nonrecurrent flowering species) and barely detected in* R. chinensis* (a recurrent flowering species) at any stage. Therefore, it can be considered that lower expression of* RTFL1c* is required for recurrent flowering of roses [92]. Iwata et al. (2012) elucidate that occurrence of TE insertion and point mutation in the* TFL1* ortholog on rose and strawberry correlate with recurrent blooming [93]. Higher expression of TFL1 in seasonal flower is associated with the repression of* LEAFY* (*LFY*) and* activating protein-1* (*AP1*), a downstream gene of FT [94]. Expression of* flowering locus T* of rose (*RoFT*) was progressively increased after floral bud formation. CONSTANS TF induces the* F*T, and, upon induction,* FT* was suggested to move from leaves to shoot apical meristem (SAM) via phloem [95–97]. Additionally,* suppressor of Ty* (*SPT*) and* delay of germination* (*DOG*)*1* are other important candidates determining recurrent blooming in roses [31].

Rose scent is a complex trait involved with hundreds of volatile molecules. Rose floral scent contains phenolic derivatives, terpenoids, and fatty acid derivatives. Several genes have been identified to be related to rose scent production. Floral scent of roses contains higher germacrene D synthase. Cyanidin and germacrene D were identified to be involved in the color and scent pathways. Sesquiterpene synthase catalyzes the production of germacrene D [98].* Phenylacetaldehyde synthase* (*PAAS*) and* phenylacetaldehyde reductase* (*PAR*) are responsible for the synthesis of 2-phenylethyl alcohol, a typical rose scent compound [99]. Anthocyanin synthesis on rose was linked with the pigmentation and volatile (scent) compounds related pathways. Anthocyanin and volatile compound have been generated by enabling the formation of MYB-bHLH-WD40 protein complex.* Orcinol-o-methyltransferase* (*RhOOMT) 1* and* 2* is responsible for synthesis of 2OMT. Alcohol acyltransferase of* R. hybrida* (*RhAAT1*) gene converts alcohol geraniol into geranyl acetate [62]. Major scent compound of European roses is 2-phenylethanol and monoterpenes [100, 101].* RhOOMTs* catalyze the orcinol to synthesize two important volatiles such as 3,5-dimethoxy toluene (DMT) and 1,3,5-metoxy benzene (TMB) biosynthesis in* R. hybrida *[62]. From the study of interhybrid cultivars of rose, DMT was concluded to come from Chinese rose, as ancient European roses such as* R. damascena* and* R. gallica* do not produce DMT [102]. Geraniol, a hydrolyzed product and its downstream monoterpene volatile metabolites, are responsible for the aroma of rose petals.* Nudix hydrolase* (*NUDX*)1 is involved in synthesis of geraniol and other geraniol-derived monoterpenes [103]. Still, there are many pathways rose scent need to be elucidated.

Genome released in rose will be helpful for decoding the metabolic networks of scent pathway, floral transition, and flowering pattern. Therefore, irrespective of complex and cumbersome heterozygous nature, interspecific hybridization can be accelerated to produce hybrid with valuable traits in rose.

## 4. Conclusions

Complete genome information of plant reduces effort and time required for conventional MAS approach. Identification and characterization of genes controlling important traits and tagging molecular markers for introgression to produce a new variety are feasible with available genome information. Along with abiotic and biotic stress resistance, several fruit quality traits can be improved with genomics-based studies. Fruit firmness is one of the desirable quality traits. It depends on the postharvest shelf life, cell turgor pressure, and intrinsic characteristics of the cell wall. Modification and turnover of the primary cell wall are required for both size and softness of fruits. New varieties/cultivars with small/larger size, good-flavored fruits, attractive color, sugar and acid levels, reduced juvenile phase, massive and constant yields, reduced susceptibility to fruit cracking, self-compatibility, and improved resistance or tolerance to disease are now feasible with the completion of the whole genome sequence.

## Figures and Tables

**Table 1:** Genome sequencing of important commercial plants belongs to the Rosaceae family.

Common name	Sample name	Chr number	Genome size		Coverage (%)	Platform	Number of genes	Repetitive sequences (Mb)	Reference
			Estimated (Mb)	Assembled (Mb)					
Apple	*Mallus* x *domestica *“Golden Delicous”	2n=2x=34	742.3	603.9	81.3	BAC + 454	57,386	362.3	Velasco et al., 2010
	*Mallus* x *domestica *“Golden Delicous” (Heterologous)		701.0	632.4	90.2	Illumina+ PacBio	53,922	382.0	Li et al., 2016
	*Mallus* x *domestica *“Golden Delicous doubled-haploid”		651.0	649.7	99.8	Illumina+ PacBio	42,140	372.2	Daccord et al., 2017

Pear	*Pyrus bretschneideri *“Dangshansuli”	2n=2x=34	512.0	501.3	97.9	BAC-by-BAC + Illumina	42,812	240.2	Wu et al., 2013
	*Pyrus communis *“Bartlett”		600.0	577.3	96.2	454	43,419	197.7	Chagné et al., 2014

Strawberry	*Fragaria vesca *ssp* vesca *acc. Hawaii 4	2n=2x=14	240.0	239.5	99.8	Illumina + 454 + SOLiD	33,264	49.8	Shulaev et al., 2010
	*Fragaria* x *ananassa *“Reikou”	2n=8x=56	692.0	697.7	100.8*∗*	454 + Illumina	64,947	328.3	Hirakawa et al., 2014
	*Fragaria iinumae*	2n=2x=14	221.0	199.6	90.3		26,411	63.2	
	*Fragaria nipponica*		208.0	206.5	99.3		21,540	52.5	
	*Fragaria nubicola*		202.0	203.7	100.8*∗*		21,053	49.9	
	*Fragaria orientalis*		349.3	214.2	61.3		17,239	56.2	

Chinese plum and Japanese apricot	*Prunus mume *“Mei”	2n=2x=16	280.0	237	84.6	Illumina	31,390	106.8	Zhang et al., 2012

Peach	*Prunus persica *“Lovell” v1.0	2n=2x=16	265.0	224.6	84.7	BAC-by-BAC	27,852	84.41	Verde et al., 2013
	*Prunus persica *“Lovell” v2.0			227.4	85.8	Illumina	26,873	-	Verde et al., 2017

Sweet cherry	*Prunus avium *“Santonishiki”	2n=2x=16	380.0	272.4	77.8	Illumina	43,349	119.4	Shirasawa et al., 2017

Rose	*Rosa chinensis *“Old Blush”	2n=2x=14	560.0	503.0	97.7	Illumina+ PacBio	36,377	341.5	Raymond et al., 2018
	*Rosa chinensis *“Old Blush” (doubled haploid –“HapOB”)		568.0±9.0	512.0	90.1 ~ 96.1	Illumina+ PacBio	44,481	279.6	Saint-Oyant et al., 2018
	*Rosa multiflora*		750	711	94.8	Illumina	67,380	417.2	Nakamura et al., 2018

*∗* The higher size of genome assembled than the estimated could be either due to limitation in the *kmer* abundance analysis or duplication occurring during the genome assembly of highly repetitive region.
